# A study protocol for a double-blind randomised placebo-controlled trial evaluating the efficacy of carrageenan nasal and throat spray for COVID-19 prophylaxis—ICE-COVID

**DOI:** 10.1186/s13063-022-06685-z

**Published:** 2022-09-15

**Authors:** Z. M. Jessop, J. Gibson, J. Y. Lim, T. H. Jovic, E. Combellack, T. D. Dobbs, K. Carter, S. Hiles, S. Islam, B. Healy, I. Humphreys, R. Eccles, H. A. Hutchings, I. S. Whitaker

**Affiliations:** 1 Reconstructive Surgery and Regenerative Medicine Research Group, Swansea University Medical School, Swansea, UK; 2 Welsh Centre for Burns and Plastic Surgery, Morriston Hospital, Swansea, UK; 3 Swansea Trials Unit, Swansea University Medical School, Swansea University, Swansea, UK; 4 Department of Microbiology, Morriston Hospital, Swansea, UK; 5 Public Health Wales Microbiology, Swansea, UK; 6 Division of Infection & Immunity, Cardiff University, Cardiff, UK; 7 School of Biosciences, Cardiff University, Cardiff, UK; 8 Patient and Population Health and Informatics, Swansea University, Swansea, UK

**Keywords:** COVID-19

## Abstract

**Introduction:**

At present, vaccines form the only mode of prophylaxis against COVID-19. The time needed to achieve mass global vaccination and the emergence of new variants warrants continued research into other COVID-19 prevention strategies. The severity of COVID-19 infection is thought to be associated with the initial viral load, and for infection to occur, viruses including SARS-CoV-2 must first penetrate the respiratory mucus and attach to the host cell surface receptors. Carrageenan, a sulphated polysaccharide extracted from red edible seaweed, has shown efficacy against a wide range of viruses in clinical trials through the prevention of viral entry into respiratory host cells. Carrageenan has also demonstrated in vitro activity against SARS-CoV-2.

**Methods and analysis:**

A single-centre, randomised, double-blinded, placebo-controlled phase III trial was designed. Participants randomised in a 1:1 allocation to either the treatment arm, verum Coldamaris plus (1.2 mg iota-carrageenan (Carragelose®), 0.4 mg kappa-carrageenan, 0.5% sodium chloride and purified water), or placebo arm, Coldamaris sine (0.5% sodium chloride) spray applied daily to their nose and throat for 8 weeks, while completing a daily symptom tracker questionnaire for a total of 10 weeks.

**Primary outcome:**

Acquisition of COVID-19 infection as confirmed by a positive PCR swab taken at symptom onset or seroconversion during the study. Secondary outcomes include symptom type, severity and duration, subsequent familial/household COVID-19 infection and infection with non-COVID-19 upper respiratory tract infections. A within-trial economic evaluation will be undertaken, with effects expressed as quality-adjusted life years.

**Discussion:**

This is a single-centre, phase III, double-blind, randomised placebo-controlled clinical trial to assess whether carrageenan nasal and throat spray reduces the risk of development and severity of COVID-19. If proven effective, the self-administered prophylactic spray would have wider utility for key workers and the general population.

**Trial registration:**

NCT04590365; ClinicalTrials.gov NCT04590365. Registered on 19 October 2020.

**Supplementary Information:**

The online version contains supplementary material available at 10.1186/s13063-022-06685-z.

## Strengths and limitations of this study

- A randomised placebo-controlled double-blind trial
- Secondary outcomes designed to improve understanding of the effects of carrageenan nasal and throat sprays on COVID-19 transmission, acquisition, severity and/or duration of resultant infection and other acute respiratory infections
- Roll out of the vaccination programme has reduced the eligible participant population from which to recruit

## Introduction

In December 2019, clusters of patients presenting with severe pneumonia of unknown origin were reported in the city of Wuhan, Hubei Provence, China. The causative organism was identified as a novel coronavirus, termed SARS-CoV-2, causing the disease known as COVID-19. In March 2020, the World Health Organization (WHO) declared a global pandemic which spread across the world despite increasingly drastic non-pharmacological interventions (social distancing, mask-wearing and lockdowns). A case fatality rate of 0.1% to over 25% has been reported [1].

SARS-CoV-2 transmission is largely via respiratory droplets (>5um, travel <1m), direct contact (touching eyes/nose/mouth) and to a lesser extent aerosolisation (smaller particles <5um, travel >1m) [2, 3]. People in prolonged close contact with an infected person are most at risk. Essential workers, especially the medical workforce, have been shown to be at a seven-fold greater risk of severe disease and hospitalisation [4–6]. Healthcare workers in patient-facing roles were three times more likely to be admitted with COVID-19 than non-patient-facing healthcare workers [7]. Furthermore, the risk of developing COVID-19 was doubled among household members of patient-facing workers, compared to those in non-patient-facing roles, when adjusted for sex, age, ethnicity, socioeconomic status, and comorbidity [7]. High viral load exposure at the onset of disease may be the cause of more serious disease in patient-facing healthcare workers [4, 7–9].

At present, vaccination and social distancing form the only mode of prophylaxis against COVID-19 [10]. Many countries have started implementing a phased vaccination rollout, however, owing to issues such as vaccine availability, achievement of mass immunisation, particularly for middle- to lower-income nations, is estimated to only be achievable by 2023 at the earliest [11]. The emergence of viral mutations [12] threatens the efficacy of our current vaccine strategy [13–19], .As such, it is imperative that other methods of transmission prevention should continue to be investigated.

Carrageenan covers a range of sulphated polysaccharides and oligosaccharides extracted from red edible seaweed. It is widely used to thicken, emulsify, and preserve food and drink, as a vegetarian and vegan alternative to gelatin, as well as in the cosmetic and pharmaceutical industry. The three commercially important carrageenans are iota-, kappa- and lambda- carrageenans, which consist of disaccharide repeating units with one, two and three sulphate ester groups respectively [20]. The in vitro antiviral activity of carrageenan was first described in 1958 when carrageenan was shown to exert a marked inhibitory effect on the growth of influenza B virus and mumps virus in embryonated chicken eggs [21]. Since then, carrageenan has shown in vitro anti-viral efficacy against a wide range of viruses [22–32]. Recently, it has been demonstrated to have in vitro antiviral activity against the novel SARS-CoV-2 [33]. In vivo clinical trial data has previously demonstrated the benefit of iota-carrageenan nasal spray in reducing the duration of symptoms of the common cold, as well as the viral load in nasal lavage, compared with saline placebo sprays [34–38]. The mechanism of action is believed to be one of preventing the binding and entry of viral particles into nasal epithelial cells [22]. In order to cause infection respiratory viruses must reach their specific host cell surface receptor by moving through respiratory fluid and mucus (10 μm depth) without any means of self-propulsion [39]. Initially, this depends on Brownian motion and eventually the positively charged virus comes close enough to the cell to be attracted via large negatively charged molecules on the cell surface known as Heparan Sulphate (HS) Proteoglycans [40]. It is thought that Carrageenan mimics the HS and can, therefore, trap the virus and prevent infection of adjacent epithelial cells [35]. Carrageenan trapped viruses are thought to be transported by mucociliary clearance to the nasopharynx, swallowed, and then destroyed in stomach acid.

SARS-CoV-2 is detected in both the nose and throat, with higher viral loads in the nose [8]. Carageenan nasal and throat spray thus has potential as an ideal prevention and early treatment for COVID-19 because of several characteristics; safety, tolerability, lack of interaction with other medication and a non-specific action against different groups of viruses including emerging variants of the same virus [35]. This protocol is written in accordance with the SPIRIT checklist.

### Research question

**Fig. 1:**
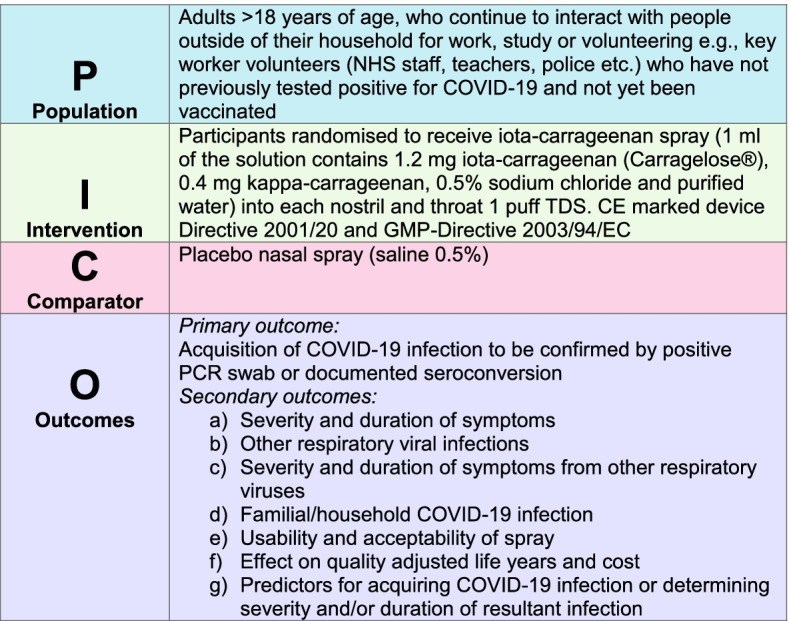
ICE-COVID research question (PICO format)

“Does carrageenan nose and throat spray prevent or reduce the severity of COVID-19?”. This is addressed using the PICO format in Fig. 1.

### Design

The design is shown in Fig. 1.

### Hypothesis

That carrageenan nasal sprays will reduce SARS-CoV-2 attachment to naso- and oropharyngeal mucosal epithelial cells and either a) prevent infection or b) reduce the severity and sequelae of resultant COVID-19 infection (through reduced effective viral infective dose exposure).

### Objectives

#### Primary objective

To determine whether carrageenan nasal and throat spray reduces the risk of COVID-19.

#### Secondary objectives

To determine:

- Whether carrageenan nasal and throat spray reduces the severity and/or duration of COVID-19 infection.
- Whether carrageenan nasal and throat spray reduces the risk of infection with other respiratory viruses
- The usability of carrageenan nasal and throat spray for long-term prophylaxis against respiratory viruses
- The effect of using the spray on quality-adjusted life years and cost-effectiveness
- Whether carrageenan nasal and throat spray reduces subsequent familial or household infection with COVID-19
- Whether any investigations or questionnaire findings in this trial offer a predictive value for acquiring COVID-19 infection or determining the severity and/or duration of resultant infection
- Any associations between symptom severity and/or duration and prognosis with COVID-19

## Methods and analysis

### ICE-COVID trial design

**Fig. 2:**
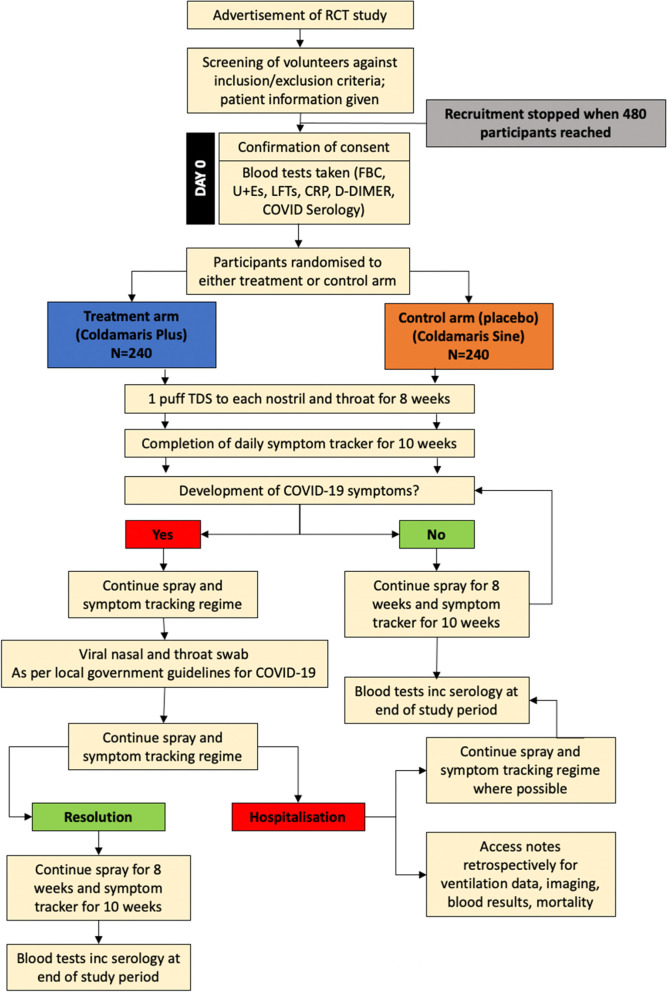
ICE-COVID trial design. The flow chart summarises the design of the ICE-COVID trial

Efficacy of iota-carrageenan endonasal and throat spray against COVID-19 (ICE-COVID) is a single-centre, phase III, double-blind, randomised placebo-controlled clinical trial. Participants will be randomly allocated to each of either the treatment arm (verum Coldamaris plus) or placebo (Coldamaris sine) arm (Fig. 2). Allocation to each group, treatment or placebo administration and data analysis will be blinded to both participant and investigator (double-blind).

### Study setting

The study will be conducted at Swansea Bay University Health Board, Swansea, UK.

### Recruitment/consent

**Fig. 3:**
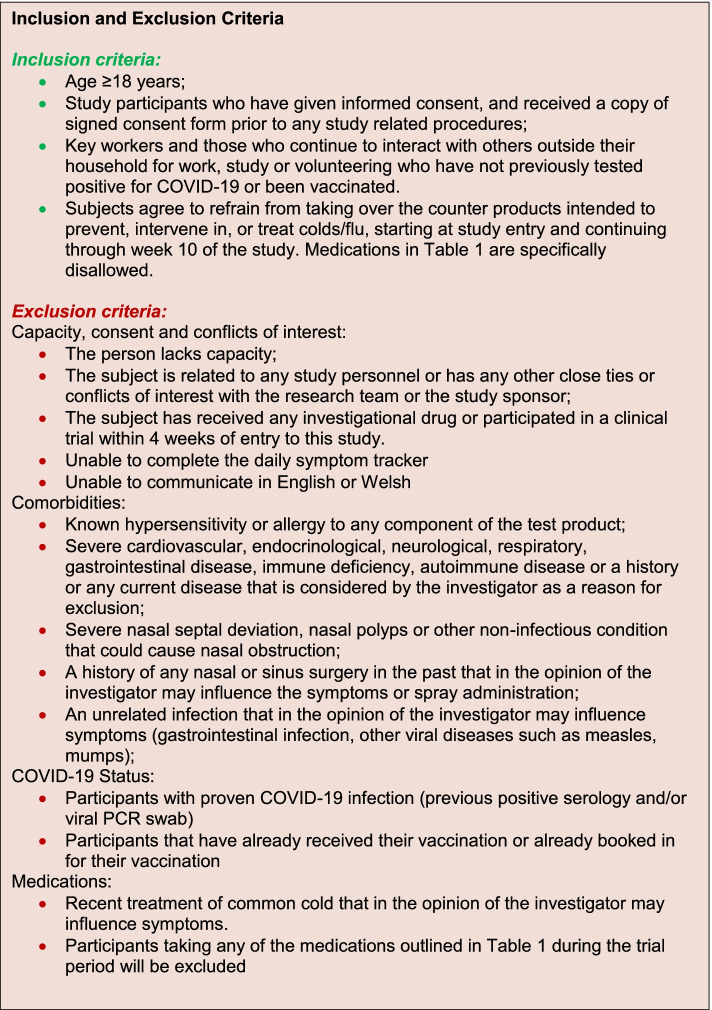
ICE-COVID inclusion and exclusion criteria

Potentially eligible participants will be identified via posters, social media and local and national press. Interested participants will be able to self-identify by contacting the research team via the research email address or study telephone. In reply, they will be given a sequential screening ID and sent an online screening questionnaire to assess their eligibility against inclusion and exclusion criteria outlined in Fig. 3 and ensure participants agree to refrain from taking disallowed medication during the trial period (Table 1). They will be entered into the screening log, on the REDCap™ database hosted by Swansea Trials Unit (https://redcap.swansea.ac.uk/), where exclusions can be documented. The screening tool will be administered electronically to minimise contact during the COVID-19 pandemic. This electronic form will be assessed by the trial team to determine eligibility for the trial.

**Table 1:** Disallowed medication during the trial period

**Disallowed co-medication**	
Antihistamines Decongestants Antitussives Combination cold products Antivirals Oral or nasal steroids All nasal sprays	

Eligible participants will be emailed the information sheet and consent form to read in advance of their face-to-face enrolment visit. Written, informed consent to participate will be obtained from all participants. During all face-to-face appointments, the recommended government guidelines will be followed on social distancing and personal protective equipment for both the research team and participants. Informed consent will be undertaken by a Good Clinical Practice (GCP) trained research nurse and recorded on the REDCap™ database prior to the participant undergoing procedures that are specifically for the purposes of the study.

### Randomisation

Following consent, baseline data will be collected, and the participants will be issued a randomisation ID which will assign them to either placebo or verum spray. This will be documented on the enrolment log. The randomised spray bottles will be stored at Swansea University, UK, for assignment to subject participants.

Randomisation sequence will be created using R 3.6.3 (R Core Team 2020) based on the pseudorandom number generation algorithm “Mersenne-Twister” using a 10-digit seed for reproduction. Participants will be assigned to the respective treatment with a 1:1 allocation and fixed block size by a statistician with no clinical involvement in the trial.

### Blinding

Blinding will occur at the site of manufacture of spray bottles, which will be assigned a randomisation number. Both the study investigators and participants will be blinded to the type of spray bottle being used. Numbered sealed blinding envelopes will be kept in Swansea University, which will only be opened if there is a severe adverse reaction to determine if the study participant received verum or placebo.

### Treatment arms/intervention

Participants will be randomly allocated to each of either the treatment arm (verum Coldamaris plus, 1 ml of the solution containing 1.2 mg iota-carrageenan (Carragelose®), 0.4 mg kappa-carrageenan, 0.5% sodium chloride and purified water) or placebo (Coldamaris sine, saline 0.5%) arm. Coldamaris plus is a CE marked device Directive 2001/20 and GMP-Directive 2003/94/EC. Participants will be instructed to administer the spray prophylactically into each nostril and throat 1 puff three times a day (TDS) according to the manufacturer’s instructions and will be asked to continue even if symptoms develop. The trial will run for a total of 8 weeks per participant, during which time the participants will be invited to complete a daily symptom tracker.

### Outcomes

#### Primary outcome

Acquisition of COVID-19 infection as confirmed by a positive PCR swab taken at symptom onset or seroconversion during the study

#### Secondary outcomes

- Severity and duration of symptoms (time taken for all symptoms to resolve, length of hospital and intensive care stay, mortality rate)
- Acquisition of non-COVID-19 respiratory viral infections, e.g. common cold or flu symptoms in non-COVID-19 positive participants
- Usability and acceptability data of nasal and throat spray as prophylaxis
- Effect on quality-adjusted life years and cost-effectiveness
- Subsequent familial/household COVID-19 infection
- Baseline haematological or questionnaire findings as predictors for acquiring COVID-19 infection or determining severity and/or duration of resultant infection
- associations between symptom severity and/or duration and prognosis with COVID-19

### Assessment, data collection and follow-up

#### Blood testing

Participants will be required to have blood tests taken to determine baseline haematology, biochemistry and clotting parameters through the following blood tests: Full Blood Count (FBC), C-Reactive protein (CRP), Urea and Electrolytes (U&E), Ferritin, Liver function tests (LFT), Lactate Dehydrogenase (LDH), D-dimer and Vitamin D level, which have been suggested as potential surrogate markers of COVID-19 severity [41–49]. These blood investigations will also be repeated at the end of the study period for each participant.

All biological samples will be analysed and stored at Swansea Bay University Health Board.

#### Seroconversion

Participants will be tested for SARS-CoV-2 antibodies using the enzyme-linked immunosorbent assay at the start and end of the study period. The presence of SARS-CoV-2 antibodies at the start of the trial indicates the previous infection with COVID-19 and the participant data will as such be excluded from the trial. Participants will be deemed to have acquired COVID-19 during the study period if they are antibody negative at the start of the study and positive for SARS-CoV-2 antibodies at the end of the study (i.e. seroconversion). This will be an essential means of detecting individuals with asymptomatic infection during the study period.

#### Quality of life data

Quality of life data will be collected using the EuroQol 5-dimension, 3-level (EQ-5D-3L) patient-reported outcome measures (PROM) [50]. This widely used, patient-completed PROM gathers generic data on health-related quality of life at specific time points when completed. The EQ-5D-3L is used on the advice of the NICE 2019 position statement, recommending its use over the new EQ-5D-5L [51]. Questionnaires will be completed at enrolment, week 4 and week 10 (study end).

#### Daily symptom tracker questionnaire

Participants will be required to complete a daily symptom tracker throughout the study period to identify the development of any symptoms that are indicative of a possible COVID-19, or other upper respiratory tract infection. The link for this will be sent to the participants daily via email.

#### Baseline assessment

Participant demographics including date of birth, gender and ethnicity will be recorded. Medical history will be recorded including current medical conditions, regular medications, and any allergies. Participants will be asked to complete a quality-of-life questionnaire: EQ-5D-3L, this will also allow for a later cost-effectiveness analysis as well as undergo the baseline blood tests outlined above. Randomisation and training in spray application will take place after baseline assessments and questionnaires have been completed, on the same day.

#### Adverse events

Adverse events are, according to the definitions, any unfavourable or unintended event affecting participants during the study. In cases of prolongation of hospitalisation, death or significant clinical sequelae, these events are defined as serious adverse events (SAEs), the occurrence of which the study sponsor (Swansea University) will be informed within 48 h of the initial observation of the event. The study treatment period is defined as the period from the first study-related nasal and throat spray administration until 14 days after the last study-related nasal and throat spray administration. Participants will be encouraged from the outset to contact the research team at the time of an event occurring by telephone or email and all details will be documented on the SAE form. The daily symptom tracker questionnaire will also provide an opportunity for symptom monitoring and the opportunity to report any symptoms that will be flagged up to the research team via a weekly report.

#### Data management

In the ICE-COVID trial, data collection is performed by trained local research staff and data entry in the REDCap database is completed contemporaneously and in a standardised fashion across two research locations in Swansea, UK. Data will be monitored via an independent data monitoring committee (DMC) using a weekly report to assess compliance with the completion of the daily symptom tracker questionnaire and identify symptomatic participants.

### Data access

Only members of the direct research team will have access to participant identifiable data. To ensure the confidentiality of samples, participants will be assigned a study identifier code which will be used to analyse data not performed at the research sites. No data will be transferred outside of the EU. Access to the system will be available for inspectors and sponsor representatives (monitors/auditors) this will enable source data verification of clinical trial subjects whilst protecting the confidentiality of non-trial patients

#### End of study treatment

Participants will be invited for an end-of-study visit and asked to bring back the empty spray bottles to be weighed as an additional measure of compliance. Participants will be asked to complete an end-of-study quality-of-life questionnaire: EQ-5D-3L, usability questionnaire as well as adverse events reporting. Participants will also undergo study exit blood tests which will include SARS-CoV2 serology.

### Ancillary and post-trial care

There is no anticipated harm and compensation for trial participation.

### Sample size and rationale for non-inferiority

We calculated the sample size based on the primary outcome measures (having COVID-19) and the respective assumed effect size in relation to the intervention (receiving iota-carrageenan spray) and the placebo nasal spray (0.5% saline). The magnitude of any effect of the nasal and throat spray on COVID-19 symptoms is unknown. However, 20.2% of healthcare professionals reported at least one symptom associated with SARS-CoV2 infection during the first wave of the pandemic, with a twelvefold increase in the risk of a positive test compared to the general population [4]. It has been recognised that key workers have been disproportionately affected by COVID-19 infections and the overall seroprevalence of SARS-CoV-2 antibodies has been found to be 24.4% in healthcare professionals [52, 53]. .Assuming an expected difference of 12.5% (i.e., 25% and 12.5% in the intervention and control group respectively), a sample of 304 participants (i.e. 152 in each arm) will provide 80% statistical power using a two-sided significance level of 5%. Assuming a lost to follow-up of 20%, a total of 380 participants (190 in each arm) will be needed.

### Statistical analysis

Data will be analysed in accordance with an intention-to-treat analysis. Each of the outcome measures will be checked for the distribution to be normal. Each of the outcome measures and important baseline covariates would be reported along with their missingness. If required appropriate imputation method would be adopted in the main analysis. All the statistical data management and analysis will be done under standard statistical software (e.g. STATA version 16 or IBM SPSS version 26).

#### Primary outcome

The primary outcome measure is a binary variable whether COVID-19 infection has been confirmed by PCR swab or not. We will perform a summary analysis reporting the outcome of the categories of two trial arms with respect to the primary outcome measure. The odds ratio and related statistical significance of the chi-square test will be reported. Following on, an adjusted analysis will be done adopting a logistic regression model. The adjustment would be done using the baseline covariates like demographics, co-morbidities (especially history of COVID-19), current medications along with baseline QoLs. The selections of the covariates would be done based on literature reviews along with discussing with the clinical co-applicants involved in this trial. This will also depend on the missing of the covariates and the selection procedure in the statistical modelling.

#### Secondary outcomes

Our approach toward the secondary outcome analysis would be mainly exploratory and report the summary outcomes of the secondary outcome measures. We will check the distributions of the data whether following normal or not. For any continuous secondary outcomes (e.g. length of hospital stay, severity and duration of symptoms) we will report the mean and standard deviation, along with the outcome of statistical tests by groups (e.g. *t*-test). The categorical outcomes will be reported using percentages and chi-square tests. In all cases, a summary of the missing data will be reported. We will also report the safety data using summary statistics by the patients and the events for each of the trial arm groups.

### Economic evaluation

An economic evaluation will be undertaken at day 70 post randomisation. The proposed secondary endpoints and methods for economic evaluation follow the guidance set out by NICE [54]. The primary economic analysis will be a cost-utility analysis expressed in terms of quality-adjusted life years (QALYs). Cost data based on the cost of a course of active ingredient spray will be obtained from the supplying company (Marinomed Biotech AG). A comparator cost analysis will be performed for the cost associated with developing COVID-19. A number of models will be developed, expressing the predicted cost of falling ill with COVID-19 but remaining at home, the cost associated with a short hospital stay and finally that associated with an admission to critical care. Treatment costs will include the cost of each scenario including healthcare professionals, equipment and infrastructure costs. Incremental cost-effectiveness ratios, incremental net monetary benefit and incremental net health benefit statistics will be calculated. A QALY in the range of £20,000–£30,000 will be considered acceptable in line with NICE guidance [54].

### Usability and compliance

In order for a new treatment or device to be useful, it must not only be efficacious but also acceptable to those that use it [55]. A nasal spray such as that trialled here needs to be easy to use, acceptable to those using it and have high compliance. Usability of the spray will be assessed with the Benefits, Satisfaction and Willingness to continue (BSW) questionnaire. This short, validated questionnaire focuses on three questions (benefit from treatment, satisfaction with treatment and willingness to continue treatment), with simple yes/no answers [56]. Compliance will be assessed with a simple question asking if the participant has been able to apply the spray every day. A free text response if they answer ‘no’ to the first question will allow qualitative exploration of reasons preventing the daily application of the spray. Data on usability and compliance will be collected at weeks 4 and 10 (study end). Further compliance data will be collected if the symptom tracker is not completed for 5 days or more.

### Management of biological samples

Viral serology will be sent to MarinomedBiotech AG. Samples will be disposed of the following analysis.

Swansea Bay University Health Board will perform an analysis of the blood tests (SARS-CoV2 antibodies, FBC, CRP, U&E, Ferritin, LFT, LDH, D-dimer, Vitamin D) and dispose of this securely following analysis. No samples will be kept for further analysis.

### Monitoring

The research project will be coordinated by a Trial Management Group (TMG), consisting of all the named investigators, Joint Clinical Research Facility (JCRF) research nurses and Swansea Trial Unit (STU) researchers. The TMG will meet remotely on a fortnightly basis to oversee the conduct and progress of the research project. An independent Data Monitoring Committee (DMC) will be established to oversee research project progress and ensure high-quality, accurate and valid data collection. Online REDCap data collection will enable real-time monitoring and inbuilt verification and validation as specific data items will link to processes on the ground. The daily tracker questionnaire will allow symptom monitoring and flag up adverse events as well as lack of compliance. An incomplete questionnaire for over 5 days will trigger a telephone call by the research team to the study participant to go through the questionnaire.

### Ethics and auditing

Ethics approval was obtained by Research Ethics Committee 6 South Wales (REC Reference 20/WA/0298; IRAS 283187) on the 18th November 2020. The trial was registered on ClinicalTrials.gov (NCT04590365) on the 19th October 2020. The results of the main trial and each of the secondary endpoints will be submitted for publication in a peer-reviewed journal. The CI, PIs and all institutions involved in the research project shall permit research project-related monitoring, audits, and REC review ensuring the study remains within the parameters set.

### Dissemination

The aim of this trial is to report definitive results regarding the effectiveness of carrageenan nasal and throat spray in protecting key workers from infection with SARS-Cov2. Dissemination of the outputs from this trial is proposed through depositing information in open access repositories prior to publication in a high-impact, open access journal and by a presentation at relevant international conferences. Given the urgency and unprecedented nature of the current situation, we will use every means available to us to ensure the results are disseminated rapidly, efficiently and effectively.

### Revision to trial design

The trial protocol was written prior to the introduction of vaccines and was initially aimed to recruit healthcare professionals who have not been previously tested positive for COVID-19. However, the success of the UK vaccination programme and prioritisation of healthcare professionals for vaccines has meant that the majority of staff were no longer eligible for the trial. Approval was therefore sought from the Sponsor (Swansea University), REC and SBUHB (NHS) R&D Office to widen the potential pool of study participants to include all key workers or those needing to interact with people outside of their household for study or work from the 11th of January 2021. The full trial protocol is available on ClinicalTrials.gov [40].

## Supplementary Information

**Additional file 1.**
